# *T*. *brucei* infections abrogate diverse plasma cell-mediated effector B cell responses, independently of their specificity, affinity and host genetic background

**DOI:** 10.1371/journal.pntd.0008358

**Published:** 2020-06-26

**Authors:** Carl De Trez, Shahid Khan, Stefan Magez

**Affiliations:** 1 Laboratory of Cellular and Molecular Immunology, Vrije Universiteit Brussel, Brussels, Belgium; 2 Department of Parasitology, Leiden University Medical Center, Leiden, Netherlands; 3 Ghent University Global Campus, Yeonsu-gu, Incheon, South Korea; University of Georgia, UNITED STATES

## Abstract

Antibody-mediated parasite killing is considered the most effective host immune response against extracellular trypanosome parasites. However, due to host-parasite co-evolution pressure, these parasites have “learned” how to hijack the host immune system via the development of immune evasion strategies. Hereby they prevent elimination and promote transmission. In the past, our group has shown that African trypanosome parasites are able to “shut down” the host B cell compartment, via the abolishment of the homeostatic B cell compartment. In line with this, we have reported that trypanosome infections result in detrimental outcomes on auto-reactive and cancer B cells. To unravel the immune mechanisms involved in these processes we adopted here a well-defined B cell vaccine model, *i*.*e*. the thymo-dependent hapten-carrier NP-CGG (4-Hydroxy-3-nitrophenylacetyl-Chicken Gamma Globulin) emulsified in Alum adjuvant. Results show that *T*. *brucei* infections abrogate the circulating titres of vaccine-induced CGG-specific as well as NP-specific IgG1+ antibodies, a hallmark of memory B cell responses in this model. This happens independently of their affinity and IFNɣ signalling. Next, we demonstrate that *T*. *brucei* infections also induce a decrease of anti-NP IgG3+ antibodies induced by the administration of NP coupled to Ficoll, a thymo-independent antigen. Confirming the non-specificity of the infection-associated immunopathology, this report also shows that trypanosome infections abolish vaccine-induced memory response against malaria parasite in BALB/c mice. Together, these data indicates that *T*. *brucei* infections impair every stages of B cell development, including effector plasma B cells, independently of their specificity and affinity as well as the host genetic background.

## Author summary

African trypanosomiasis is a fatal infectious disease caused by an extracellular parasite of the *Trypanosoma brucei* species affecting both human and livestock. The most effective immune response against this pathogen involves the production of antibodies by B cells. However, experimental trypanosomiasis model in mice has demonstrated that this parasite has evolved multiple immune evasion strategies targeting B cells. For instance, trypanosomes abolish homeostatic B cell development, the adaptive protective response against unrelated antigens as well as the progression of B-cell mediated arthritis and multiple myeloma. Here, we demonstrate that infection of resistant C57BL/6 mice impairs the development of both thymo-dependent and -independent humoral response using the well-characterized hapten-carrier NP-CGG (4-Hydroxy-3-nitrophenylacetyl-Chicken Gamma Globulin) emulsified in Alum adjuvant and NP-Ficoll models, respectively. This occurs independently of antigen specific B cell affinity and the pro-inflammatory IFNɣ cytokine signalling. Finally, trypanosoma abolishes the vaccine-induced memory response against another life-threatening parasite, namely malaria, in *Trypanosoma brucei* susceptible BALB/c mice. In summary, African trypanosomiasis abrogates diverse plasma cell-mediated effector B cell responses, independently of their specificity, affinity and host genetic background.

## Introduction

Extracellular protozoan African trypanosomes (AT) parasites are the causative agent of the *Sleeping Sickness* in humans and *Nagana* in cattle and livestock [1,2]. The immune response against AT parasites is mainly mediated by B cells and their production of antibodies [3]. B cells start their development in the bone marrow from the common lymphoid progenitor stage, which further give rise to several developmental stages of pre-pro-B, pro-B, pre-B and eventually immature B cells [4]. At this stage, immature B cells migrate out of the bone marrow and arrive via the circulation in the spleen as transitional B cells. These latter further differentiate into mature marginal zone (MZ) B cells or follicular (Fo) B cells [5].

In order to counteract B cell-mediated immune responses, African trypanosomes have evolved different immune-evasion strategies mainly focusing on the dampening of host B cell activation and antibody-mediated response. For example, AT parasites are able to abolish B cell homeostasis in lymphoid organs, such as the bone marrow and spleen. They also cause the downregulation of detrimental B cell responses, e.g. autoimmune and malignant B cells [6–8].

Throughout the years, many murine B cell models have been developed. From these studies, two main classes of B cell responses have emerged, the thymo-dependent (TD) and -independent (TI) models that either do, or do not, rely either on the presence of T cells. The thymo-dependent hapten-carrier (4-hydroxy-3-nitrophenyl)-acetyl-chicken gamma globulin (NP-CGG) emulsified in Alum adjuvant constitutes one of the most studied and well-characterized model to study humoral/memory responses. For example, this model has been used for decades to investigate the development of high affinity anti-NP IgG1^+^ antibodies that arose from T cell-mediated proliferating B cells, that have undergone affinity maturation and selection in germinal center B cells [9]. In contrast, the thymo-independent NP hapten coupled to Ficoll, a high molecular weight polysaccharide (NP-Ficoll) model, induces the rapid production of low affinity anti-NP IgM^+^ and IgG3^+^ antibodies by MZ B cells in the absence of T cells [10].

Here we tested the capacity of the AT parasites to impede the development of various antibody-mediated B cell responses. In addition, we addressed the unbiased functional effect of AT parasite induced B cell immunopathology using an anti-malaria coinfection model. Together the data presented here demonstrate the general detrimental effect that trypanosome have on the wider range of B cell populations in their host.

## Material and Methods

### Ethics statement

All experiments, maintenance and care of the mice complied with the European Convention for the Protection of Vertebrate Animals used for Experimental and Other Scientific Purposes guidelines (CETS n° 123) and were approved by the Ethical Committee for Animal Experiments (ECAE) at the Vrije Universiteit Brussel (Belgium) (Permit Number: 10-220-13).

### Mice, parasites, immunization and treatment

Wild-type and IFNɣ receptor-deficient C57BL/6 mice (8 to 10 weeks old, Janvier, France) were immunized with 50μg of (4-hydroxy-3-nitrophenyl)-acetyl coupled to chicken gamma globulin (NP_36_-CGG) (Biosearch Technologies, CA, USA), emulsified in Imject Alum (Thermo Scientific, IL, USA) or with 30μg of NP_67_-Ficoll (Biosearch Technologies, CA, USA) via intraperitoneal administration. A soluble boost 30μg of NP_36_-CGG in PBS was administered 65 days post-immunization.

Clonal pleomorphic *T*. *brucei* AnTat 1.1E parasites have originally been obtained from Prof. N. Van Meirvenne (Institute for Tropical Medicine, Belgium). Infected blood aliquods (mouse) are stored at −80°C. Female C57BL/6 mice (8- to 10-weeks old, Janvier, France) were infected with 5000 AnTat1.1E trypanosomes (intraperitoneally (i.p.)) at day 20 and day 6 post NP-CGG and NP-Ficoll administration, respectively.

*P*. *berghei* parasites genetically deficient in both *plasmepsin-4* and *bergheipain-2* (*pm-4*/*bp2 dko P*. *berghei*) were used in collaboration with Shahid Khan, The Netherlands. BALB/c mice were vaccinated with 10^5^ *pm-4*/*bp2 dko P*. *berghei*-infected red blood cells (iRBCs) i.p.. Six months later, vaccinated mice were either left untreated or infected with 5000 AnTat1.1E trypanosomes. Three weeks post-*T*. *brucei* infection, all mice were treated i.p. with diminazene aceturate (Veriben, 40 mg/kg, Sigma Aldrich, St. Louis, MO, USA) in water, before being i.p. rechallenged with wild-type *P*. *berghei* ANKA parasites expressing a GFP-luciferase fusion protein under the transcriptional control of the *ama-1* promoter (10^5^ infected RBCs) that allows the monitoring of parasite load in blood, distribution and patterns of schizont sequestration in live mice by real time *in vivo* imaging.

Blood was drawn via the tail at different time-points for serological analyses.

### Enzyme-linked immunosorbant assay (ELISA) technique

96 well MicroWell MaxiSorp flat bottom plates (Sigma-Aldrich) were coated with 2μg/ml of NP26-BSA, NP4-BSA (Biosearch Technologies, CA, USA) and CGG (Jackson ImmunoResearch) in PBS and incubated overnight at 4°C. The following day the plates were washed with PBS-Tween20 0.1% and blocked with PBS/BSA 1% for 3 hours at room temperature. After removal of blocking solution, serial serum dilutions in PBS/BSA 0.1% were added to the plates and incubated overnight at 4°C. The next day, plates were washed with PBS-Tween20 0.1% and incubated with anti-mouse IgG1, IgG3 and IgM isotypes coupled to Horse Radish peroxydase (Lo-Imex, Louvain, BE) for 2 hours at 37°C. After this incubation, plates were washed and revealed using a TMB kit (BD Biosciences). The reaction was stopped using a 2M sulfuric acid solution and the plates were read at 450nm.

### Statistics

The GraphPad Prism 4.0 software was used for statistical analyses (Two-way ANOVA or student *t*-test). Values are expressed as mean ± SEM. Kaplan-Meier survival curve was analyzed by means of the Log-Rank method. Values of p≤0.05 are considered statistically significant, where * = p≤0.05, ** = p≤0.01 and *** = p≤0.001. When low (NP_26_) and high (NP_4_) affinity antibodies are compared on the same graph, * and * are for uninfected (square) and infected (triangle) mice, respectively. When wild-type and IFNɣ receptor-deficient mice are compared on the same graph, * and * are for wild-type and IFNɣ receptor-deficient mice, respectively.

## Results

### *T*. *brucei* infection affects conventional T cell-dependent humoral memory responses

**Fig 1:**
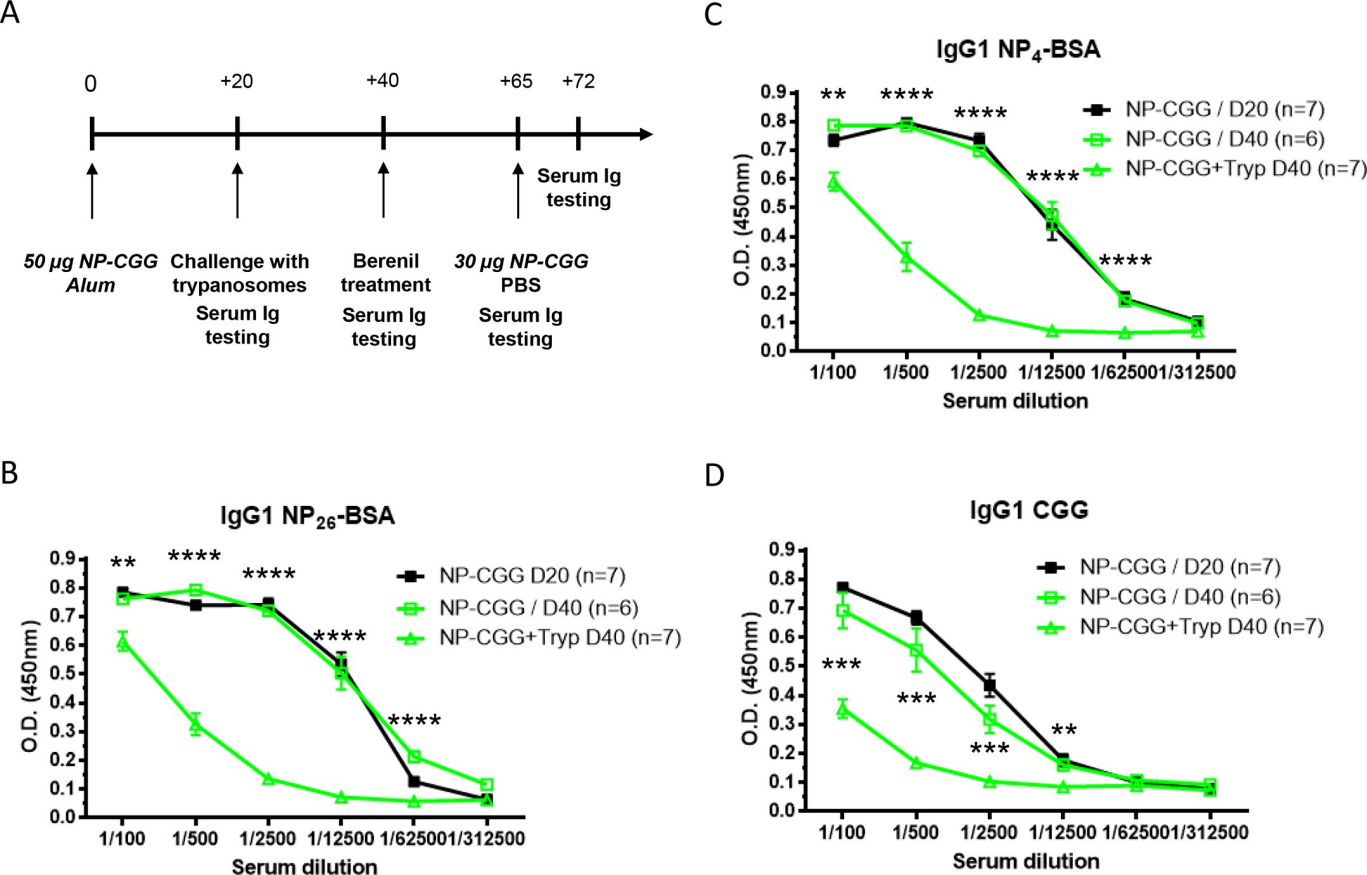
*T*. *brucei* infection dampens the primary humoral response against thymo-dependent antigens. C57BL/6 mice were immunized with NP-CGG emulsified in Alum on day 0. Twenty days later serum was collected and mice were randomly divided in two groups. One group of vaccinated mice was infected with *T*. *brucei* and the other one was left uninfected. At day fourty both groups were treated with Veriben, and on and day sixty-five they were both rechallenged with soluble NP-CGG, respectively (**A**). Serial serum dilution levels of anti-NP IgG1 Abs on NP_26_-BSA and NP_4_-BSA (**B,C**) and anti-CGG IgG1 Abs (**D**) were measured by ELISA on day 20 (before infection, black lines) and day 40 in uninfected (before Berenil treatment, open green squares) and infected (open green triangles) mice. Graphs show the mean ± SEM from at least n = 6 mice per group and the data are representative of two independent experiments.

Previous results by our group have demonstrated that *T*. *brucei* infection abrogate vaccine-induced protective memory response against a non-related pathogen, such as *Bordetella pertussis*, using a well-established human diphtheria, tetanus, and *B*. *pertussis* (DTPa) vaccination model in mice [11]. However, a clear link between the abrogation of the memory response and the disappearance of antigen-specific memory B cells has not been established. Therefore the impact of trypanosomosis on the maintenance of antigen-specific IgG1+ memory B cells using a well-characterized thymo-dependent (TD) antigen, *i*.*e*. (4-hydroxy-3-nitrophenyl)-acetyl coupled to chicken gamma globulin (NP-CGG) emulsified in Alum, was investigated. In order to avoid any impact on *T*. *brucei* parasite on the development of germinal center leading to memory response development, mice were vaccinated approximately 3 weeks before infection (Fig 1A). Before infection, anti-NP IgG1 antibody titers were analyzed on a NP_26_-BSA antigen binding ELISA using sera from vaccinated mice (Fig 1B–black line). Forty days post-vaccination, corresponding to 20 days post-infection, the IgG1 titers of circulating anti-NP specific antibodies were analyzed again in vaccinated-infected mice as well as control vaccinated mice that were not exposed to a trypanosome challenge. The obtained data indicates that the ongoing infection resulted in a drastic reduction of NP-CGG specific antibody titers (Fig 1B–green lines). An identical trend is seen when NP-CGG specific antibody titers are measured in a NP_4_-BSA antigen binding assay (Fig 1C). The comparison of the binding capacity of these circulating anti-NP specific IgG1 antibodies on two differentially haptenated NP-BSA substrates, namely NP_26_-BSA vs. NP_4_-BSA, provide an indication of antibody-target binding affinity. Indeed, while both high and low affinity Abs bind to long-haptenated NP_26_-BSA (Fig 1B), mainly high affinity Abs (but not low affinity Abs) bind to short-haptenated NP_4_-BSA (Fig 1C). Hence, the presented data demonstrates that that *T*. *brucei* infections reduce anti-NP-specific IgG1 antibodies independently of their affinity. This antibody suppression effect by *T*. *brucei* infections also affects anti-carrier CGG IgG1 Ab titers, reinforcing the unbiased depleting capacity of AT infection (Fig 1D).

**Fig 2:**
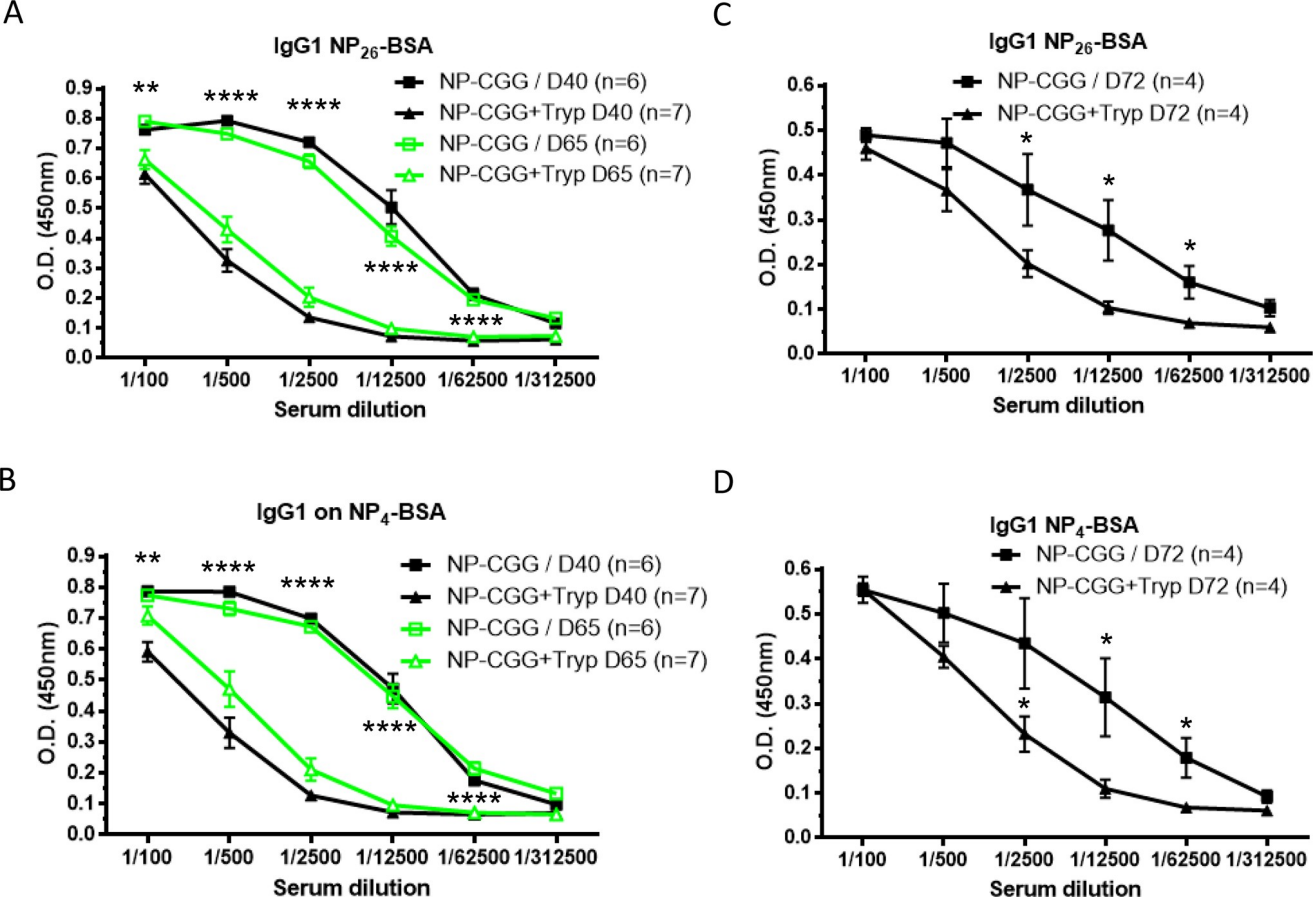
Chemotherapy and soluble antigenic rechallenge do not restore humoral response against thymo-dependent antigens following *T*. *brucei* infection. C57BL/6 mice were immunized with NP-CGG emulsified in Alum on day 0. Twenty days later, on group of vaccinated mice was infected with *T*. *brucei* and the other one was left uninfected (**A,B,C,D**). At day fourty and sixty-five, both groups were treated with Veriben and rechallenged with soluble NP-CGG, respectively. Serial serum dilution levels of anti-NP IgG1 Abs on NP_26_-BSA (**A**,**C**) and NP_4_-BSA (**B,D**) were measured by ELISA on day 40 (before Berenil treatment, black lines) and day 65 in uninfected (black and green squares, respectively) and infected (black and green triangles, respectively) mice (**A,B**) and on day 72 (7 days post soluble rechallenge; squares represent uninfected mice and triangles represent infected mice) (**C,D**). Graphs show the mean ± SEM from at least n = 6 mice per group and the data are representative of two independent experiments.

Experimental infections of C57BL/6 mice with pleomorphic *T*. *brucei* parasites usually results in a fatal outcome after 4 to 5 weeks. Hence, if longer-term immune responses are to be studied, a curative drug intervention step is needed. Here, we implemented a set-up in which the trypanocidal diminazine diaceturate (Veriben) drug was administered at day 40 post-vaccination in order to clear infection. This treatment does not restore the levels of low and high affinity anti-NP IgG1 antibodies at day 65 post-vaccination (day 25 post cure) using the T cell dependent NP-CGG model (Fig 2A and 2B–green lines). However, the administration of a NP-CGG saline boost at day 25 post-cure (day 65 post-vaccination) allows the levels of anti-NP IgG1 antibodies to increase in previously infected mice within a week (D72), albeit the observed titers do not reach the same levels as those observed in uninfected mice. The same recover trend is observed for general anti-NP titers measured in a NP_26_-BSA antigen binding ELISA (Fig 2C) as for the high affinity anti-NP_4_-BSA antibody titers (Fig 2D). In order to make sure that the soluble NP-CGG administration was indeed boosting the remaining anti-NP specific B cells at day 65 post-vaccination and not inducing a primary response due to the presence of aggregates and/or lipopolysaccharide contamination, an add-on control experiment was performed (S1 Fig). This latter confirms that soluble NP-CGG administration does not induce any specific anti-NP IgG3 response compared to NP-Ficoll.

**Fig 3:**
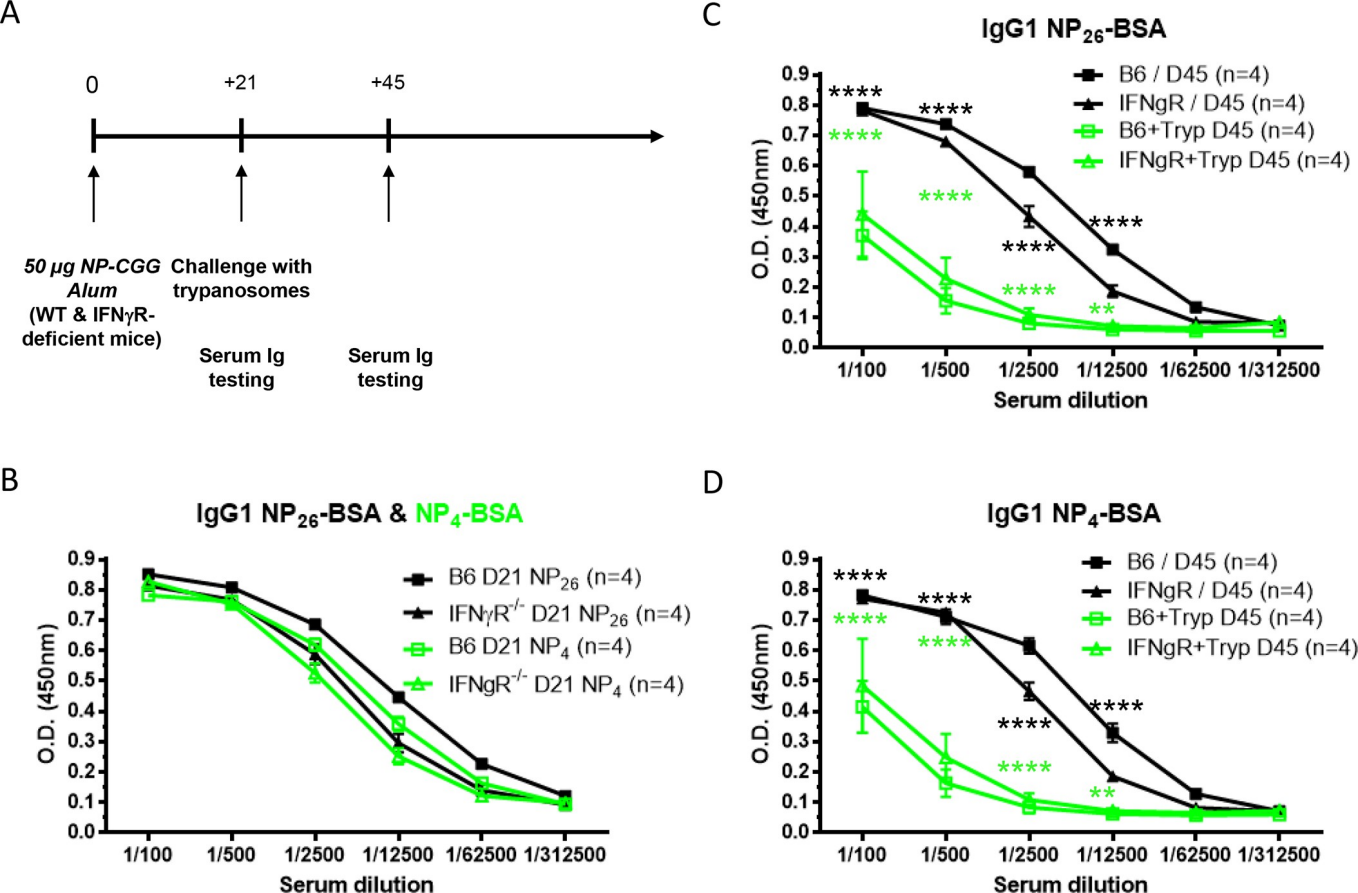
*T*. *brucei* infection dampens the primary humoral response against thymo-dependent antigens independently of IFNɣ signaling. IFNɣR-deficient and wild-type C57BL/6 mice were immunized with NP-CGG emulsified in Alum on day 0. Twenty-one days later, one group of vaccinated IFNɣR-deficient and wild-type C57BL/6 mice mice was infected with *T*. *brucei* and the other one was left uninfected (**A**). Serial serum dilution levels of anti-NP IgG1 Abs on NP_26_-BSA (black line) and NP_4_-BSA (green line) were measured by ELISA on day 21 before infection in IFNɣR-deficient (triangle) and wild-type (square) C57BL/6 mice (**B**). Serial serum dilution levels of anti-NP IgG1 Abs on NP_26_-BSA (**C**) and NP_4_-BSA (**D**) were measured by ELISA on day 45 (24 days post-infection) in uninfected (black lines) and infected (green lines) IFNɣR-deficient (triangle) and wild-type (square) C57BL/6 mice. Graphs show the mean ± SEM from at least n = 6 mice per group and the data are representative of two independent experiments.

Previous results by our laboratory have identified a role of the pro-inflammatory cytokine IFNɣ in the early onset of follicular B cell apoptosis after *T*. *brucei* infection [12]. Here, using a similar experimental approach, C57BL/6 wild-type (WT) and IFNɣ-receptor (IFNɣR)-deficient mice were vaccinated with NP-CGG in Alum adjuvant and three weeks post-vaccination, antibody titers were tested (Fig 3A). Both WT and IFNɣR-deficient mice express similar levels of circulating high (NP_4_-BSA) and low affinity (NP_26_-BSA) anti-NP specific IgG1 antibodies at day 21 post-vaccination (Fig 3B). Infection with *T*. *brucei* at day 21 post-vaccination induces a similar decrease of low and high affinity anti-NP IgG1 antibodies in both WT and and IFNɣR-deficient mice at day 45 post-vaccination (day 24 p.i.) (Fig 3C and 3D). These results suggest that the decrease of circulating anti-NP specific IgG1 levels occurred independently of IFNɣ signaling.

In summary, trypanosomosis affects T cell dependent memory immune responses as measured by the IgG1^+^ titers of both specific anti-hapten (NP) and anti-carrier (CGG) antibodies. This happens independently of their affinity for the NP hapten and the presence of the pro-inflammatory IFNɣ cytokine.

### Trypanosomosis hinders T cell-independent (TI) type 2 (TI-2) humoral response

**Fig 4:**
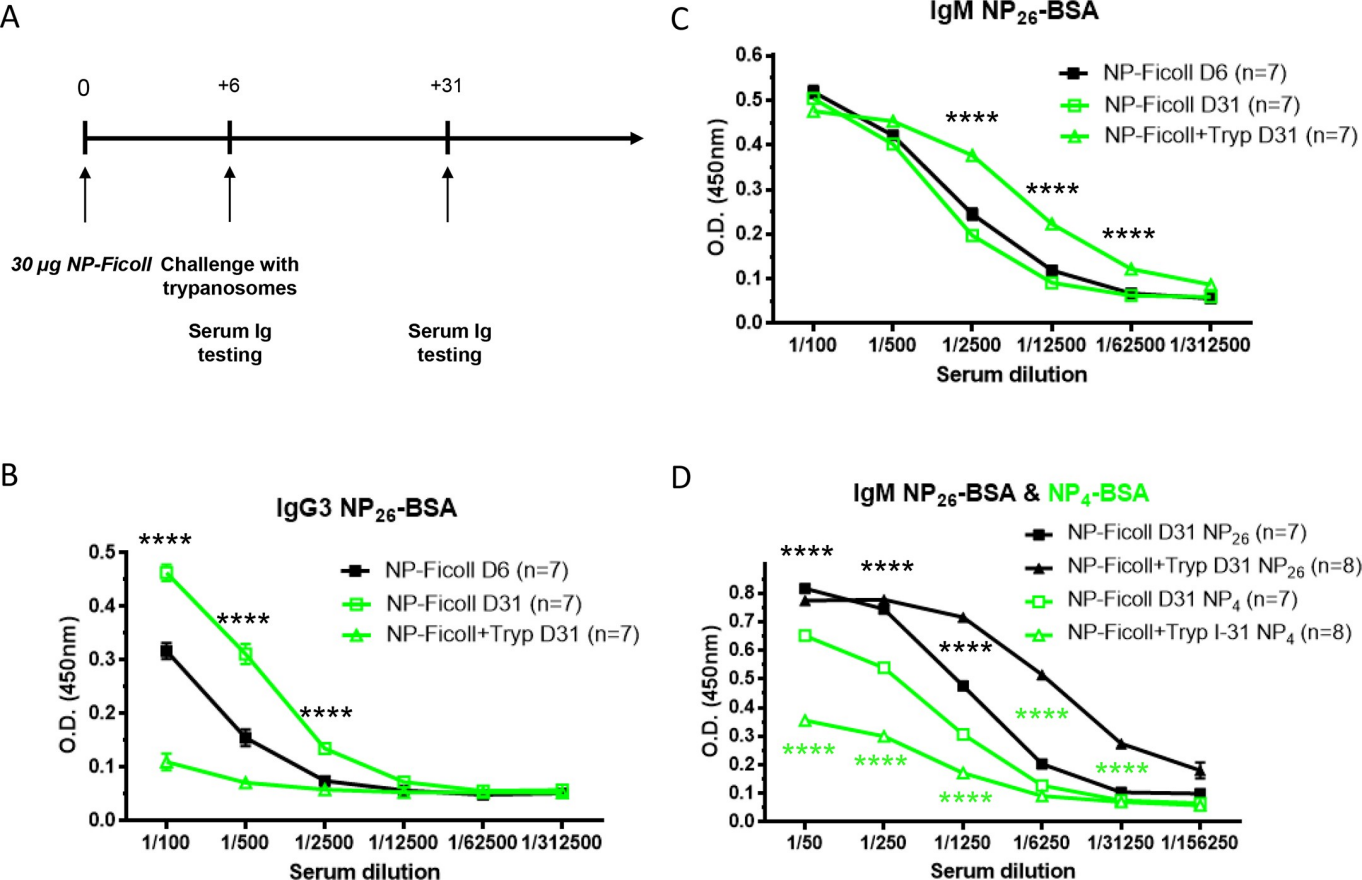
*T*. *brucei* infection hampers the humoral response against thymo-independent antigens. C57BL/6 mice were immunized with NP-Ficoll on day 0. Six days later, one group of vaccinated mice was infected with *T*. *brucei* and the other one was left uninfected (**A**). Serial serum dilution levels of anti-NP IgG3 Abs (**B**) and anti-NP IgM Abs (**C**) on NP_26_-BSA were measured by ELISA on day 6 (before infection, black lines) and day 31 in uninfected (open green squares) and infected (open green triangles) mice. Serial serum dilution levels of anti-NP IgM Abs (**D**) on NP_26_-BSA (black lines) and on NP_4_-BSA (green lines) were measured by ELISA on day day 31 in uninfected (squares) and infected (triangles) mice. Graphs show the mean ± SEM from at least n = 6 mice per group and the data are representative of two independent experiments.

Trypanosome infections have been described in the past to be characterized by rapid T cell independent antibody secretion, even preceding the first peak of infection that occurs around day 4–5 post challenge [13–15]. This signals an effort by the host to contain infection before systemic damage is being done by the parasite. Hence, we next investigated if trypanosomes are able to modulate humoral responses against thymo-independent (TI) antigens. Immunization with the same NP hapten, coupled to a TI carrier such as Ficoll, a large polysaccharide with repeating antigenic determinants engaging multiple receptors on B cell surface is considered as a typical TI-2 antigen [16]. In contrast to the TD NP-CGG antigen used in the experiments outlined above, the immune response against NP-Ficoll is dominated by NP-specific IgM and IgG3 antibodies, which reach their maximum in the serum round day 7 and 14, respectively. In the experimental setup outlined in Fig 4, mice were vaccinated once with NP-Ficoll and challenged six days later with *T*. *brucei* parasites. A control group received the vaccine without subsequent infection (Fig 4A). Vaccine induced antibody titers were measured using the same NP_26_-BSA and NP_4_-BSA antigen ELISAs as used previously. At day 31 (day 25 post infection), a significant drop in the NP-specific IgG3 antibody titers is observed in the infected group, whereas an antibody titre increase is observed in the vaccinated non-infected group (Fig 4B). Surprisingly, the levels of NP-specific IgM antibodies are increased in the vaccinated infected group compared to the uninfected group (Fig 4C). It should be noted however that other data obtained in experimental trypanosome models have shown the occurrence of infection-induced cross-reactive antibodies against trinitrophenyl (TNP) [17]. This can explain our results as the IgM^+^ antibodies detected here show some cross-reactivity with the highly haptenated NP_26_-BSA coating antigen, whereas no cross-reactivity was observed for IgG3^+^ antibodies (S2 Fig). This only becomes apparent when comparing anti-NP-BSA IgM titers on both NP_26_- and NP_4_-BSA at day 31 in infection (Fig 4D). These results indicate indeed that in infected mice, a substantial drop (~500-fold) in the circulating levels of high affinity (NP_4_-BSA) anti-NP IgM titers (open green triangle) is observed when compared with low affinity (NP_26_-BSA) anti-NP IgM levels (closed black triangle). In contrast, the difference in uninfected mice between the levels of high (open green square) and low (close black square) affinity anti-NP IgM is less than five-fold.

Together, these results demonstrated that *T*. *brucei* infection reduces both NP-specific IgM and IgG3 levels during the development of a TI-2 humoral response against NP-Ficoll, and that trypanosome parasites are able to induce low affinity cross-reactive anti-NP IgM antibodies.

### Abolishment of vaccination-induced protection against malaria following *T*. *brucei* infection occurs independently of host genetic background

**Fig 5:**
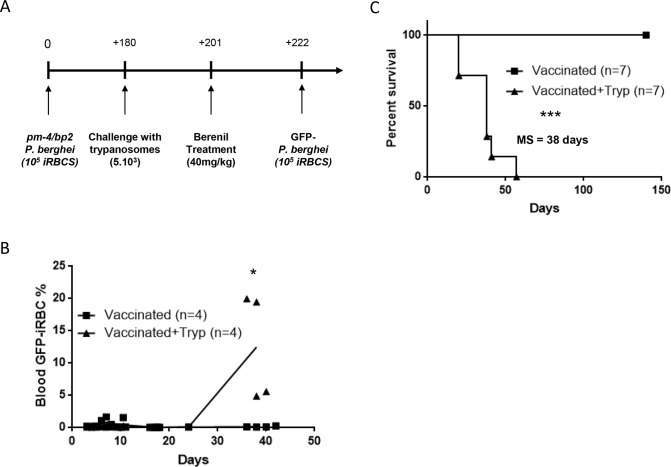
*T*. *brucei* infection blunts the existing vaccine-induced memory response aginst malaria parasites. BALB/c mice were immunized with *pm-4/bp2*-deficient *P*. *berghei* on day 0. One hundred and eighty days later, one group of vaccinated mice was infected with *T*. *brucei* and the other one was left uninfected. At day two hundred and one and two hundred and twenty-two, both groups were treated with Veriben and rechallenged with GFP-*P*. *berghei*, respectively (**A**). Evolution of GFP^+^ infected red blood cells (**B**) and survival (**C**) of infected (triangle) and uninfected (square) vaccinated mice. Graphs show the mean and scattered plots from at least n = 4 mice per group and the data are representative of two independent experiments.

In the past, we have shown the detrimental effect of experimental trypanosomosis on vaccine recall responses, using the C57BL/6 mouse DTPa model [18]. To expand on this concept, and to investigate how general the memory destruction is upon trypanosome challenge, we included in this study a malaria vaccine model in BALB/c mice. BALB/c mice infected with wild-type *Plasmodium berghei* (*P*. *berghei)* parasites succumb from severe anemia and uncontrolled parasitemia approximately two-to-three weeks post-infection [19]. In contrast, when BALB/c mice are challanged with *P*. *berghei* parasites that are genetically deficient in both a merozoite surface protein, *msp-7*, cells and a plasmepsin-4 (*pm-4*), long lasting protective and cross-reactive immunity against a lethal rechallenge with wild-type *P*. *berghei* and *P*. *yeolii* parasites, is obtained [20,21]. This protective immunity is acting through antibody-mediated parasite clearance in the spleen [21]. Therefore, in order to assess the impact of *T*. *brucei* infection on this B cell-mediated protective immunity, BALB/c mice were vaccinated with *pm-4*/*bp2 dko P*. *berghei* parasites [22] (Fig 5A). Six months later (~day 180), mice were infected with *T*. *brucei*, while others were kept as uninfected controls. Three weeks into the infection, all mice were treated with Veriben (~day 201), using a dose that cured the infection in trypanosome challenged mice. Twenty-one days post-Veriben treatment (~day 222), all the vaccinated mice were challenged with wild-type GFP-luciferase-expressing *P*. *berghei* parasites. Results demonstrate that vaccinated and *T*. *brucei*-exposed BALB/c mice all were susceptible to the *P*. *berghei* challenge (Fig 5B) succumb to the complication of the infection with 2 months following the malaria exposure (Fig 5C). In contrast, vaccinated BALB/c mice that did not experience an intermediate *T*. *brucei* infection that are still all alive 5 months later and showed complete anti-malaria vaccine induced protection. These results confirmed that a *T*. *brucei* infection is capable of abrogating the efficacy of a B cell-mediated vaccine against other parasites such as malaria, independently of the host genetic background.

## Discussion

In order to establish a chronic infection in its host and to promote its transmission, African trypanosomes have developed multiple strategies to evade host immune B cell response. Among these, one of the most studied mechanisms is the antigenic variation process. This mechanism allows the parasite to continuously change its antigenic coat in order to evade its antibody-mediated elimination [23,24]. Other data have shown that African trypanosomes can also directly target B cells, most likely by inducing their apoptosis in both the bone marrow and spleen [6,18]. On top of the parasite impact on B cell homeostasis, our previous results have shown that African trypanosomes can suppress the development of existing detrimental B cells, such as autoimmune and cancer B cells. For example, *T*. *brucei* parasites blocks the development of B cell-mediated collagen-induced arthritis in DBA prone mice, most likely by affecting the survival of anti-type II collagen specific antibody [8]. As a result, the use of diminazine diaceturate (Berenil) to treat trypanosomosis, restores the clinical signs of arthritis, most likely by restoring specific autoimmune antibody levels. Also, African trypanosomes impact the development of multiple myeloma, which are malignant plasma cells, by inducing their intrinsic *in vivo* apoptosis, which was associated to the downregulation of the endogenous unfolded protein response [7]. Using a well-characterized TD, namely NP-CGG emulsified in Alum adjuvant, and TI, NP-Ficoll, model, we investigated here if *T*. *brucei* parasites were able to affect both high and low antigen-specific B cells. Infection of NP-CGG immunized C57BL/6 mice demonstrates a similar impact of the parasite on both high and low affinity NP-specific IgG1 antibody levels in serum as well as a negative impact on the systemic levels of anti-CGG antibodies. Here, the administration of Veriben did not restore the primary systemic levels of anti-NP IgG1 antibodies. However, the secondary response induced by the administration of NP-CGG in phosphate buffer restores some levels of both high and low affinity anti-NP IgG1 antibodies, but not to the same levels as in uninfected C57BL/6 mice. This results contrasts with the effect of Berenil within the context of collagen-induced arthritis model [25]. These results suggest that in case of vaccination, irreversible partial destruction of the memory cell compartment took place during the trypanosome infection, resulting in an inability to deliver a full recall response upon reencounter of the antigen. In our previously published collagen-induced arthritis model, the partially destroyed auto-immune response was allowed to resurface after the suppressive effect of the trypanosome infection had been removed.

Previous results by our laboratory have identified a role of the pro-inflammatory cytokine IFNɣ in the early onset of follicular B cell apoptosis following trypanosomosis [12]. This cytokine was also been implicated in other immunopathologies, such anemia of inflammation [26], but also in the resistance to African trypanosome infection [27,28]. Using the NP-CGG in Alum vaccination model, we demonstrate that the impact of the infection on circulating anti-NP IgG1 antibodies is however independent of IFNɣR signalling, which is an agreement with another study investigating the mechanism implicated in B cell death in after *T*. *brucei* infection [29].

*T*. *brucei* infection of C57BL/6 mice inoculated with the TI antigen NP-Ficoll also has a drastic negative impact on the systemic levels of anti-NP IgG3 antibodies 25 days post-infection (day 31 post NP-Ficoll) compared to uninfected mice. In contrast, the level of circulating anti-NP IgM antibodies increased in infected vs. uninfected mice. This result could be explained by the generation of cross-reactive anti-NP antibodies following *T*. *brucei*-induced polyclonal B activation, as a clear difference between systemic levels of high and low anti-NP IgM is observed in infected mice. Similar results were already described in the context of *T*. *vivax* infection [17]. These results might be linked to the fact that TI antigens, such as type 2 TI antigen NP-Ficoll, mainly activate MZ B cells to produce IgG3 and IgM antibodies [30,31] and that these latter are depleted following infection [18].

Together, these results demonstrate the African trypanosomes infection can impact the levels of systemic levels of anti-NP IgG3 and IgM antibodies in response to the administration of a type 2 TI antigen, such as NP-Ficoll.

Finally, previous results from our laboratory have shown that *T*. *brucei* infection can abrogate the vaccine-induced protective immune response against a non-related pathogen such as *B*. *pertussis* [18]. However, the direct effect of African trypanosome infection on B cells was not defined within this experimental set-up. Therefore, using an experimental B cell-dependent model of vaccination against malaria, we showed now that African trypanosome infection was able to abolish the B cell-mediated protection against live malaria challenge in susceptible mice. In various animal models, other pathogens, such as Salmonella bacteria and measles virus infection, were also able to interfere with the development of efficient humoral immune responses [32,33]. If potentially applicable to humans, this detrimental effect of African trypanosomes could have important implications in endemic areas, in particular when considering a future anti-malaria vaccine application. While such vaccine is not available yet, there is an ongoing effort to develop such intervention strategy that could help the global eradication of malaria. Taken our results, it will be important to ensure that such approach is also effective in HAT endemic areas, which largely overlap with malaria endemic areas in sub-Sahara Africa. In humans, studies that address the impact of trypanosome infections on host B cell immunity and vaccine induced memory are virtually absent. One publication reported a significant lower concentrations of anti-measles Abs in vaccinated HAT patients vs. vaccinated HAT-negative individuals [34]. The differences between these two groups of patients persisted even seven months post-trypanocidal treatment. However, the impact of this decrease was not tested during a new encountering with the causative virus. Neither was it tested which proportion of the remaining anti-measles titres resulted from parasite-mediated polyclonal low-affinity Ab activation. More recently, human measles infection was also shown to compromise immune memory to previously encountered pathogens due to depletion of previously expanded B memory clones [33]. In addition, malaria parasite and HIV are also able to decrease the potential of memory B cells to differentiate into plasma B cells [35,36]. At this stage however, the exact mechanisms leading to the abrogation of different specific humoral responses following *T*. *brucei* infection remain to be unveiled, as both direct (e.g. decreasing half-life of specific antibodies in combination with B cell apoptosis) as well as indirect events (e.g. a modulation of lymphoid microenvironment or any other secondary infection-associated effector mechanism that could affect memory B cell functions), could be involved.

Together, these results confirm a detrimental role of African trypanosomes infection on B cell homeostasis and their effector functions and these data could have huge implications in terms of vaccine design and strategy in endemic areas.

## Supporting information

S1 Data This prism file summarizes all the raw data required to design the different figures presented in this article. (PZF)

S1 Fig Impact of soluble NP-CGG administration on primary specific anti-NP response. C57BL/6 mice were immunized with NP-Ficoll (square) or NP-CGG (triangle) on day 0. Serial serum dilution levels of anti-NP IgG3 Abs on NP_26_-BSA were measured by ELISA on day 7. Graphs show the mean ± SEM from at least n = 4 mice per group and the data are representative of one independent experiment.
(TIF)

S2 Fig Cross-reactive potential of anti-*T*. *brucei* antibody. C57BL/6 mice were infected with *T*. *brucei*. Serial serum dilution levels of anti-NP IgM (filled square) and IgG3 (filled triangle) Abs from pooled (n = 3) day 6-infected and uninfected mice were measured. The data are representative of two independent experiments.
(TIF)
