# Supplementary figures and images for: T. brucei infections abrogate diverse plasma cell-mediated effector B cell responses, independently of their specificity, affinity and host genetic background
Source: PLoS Negl Trop Dis. 2020 Jun 26;14(6):e0008358. doi: 10.1371/journal.pntd.0008358 (PMC7347239; doi:10.1371/journal.pntd.0008358)

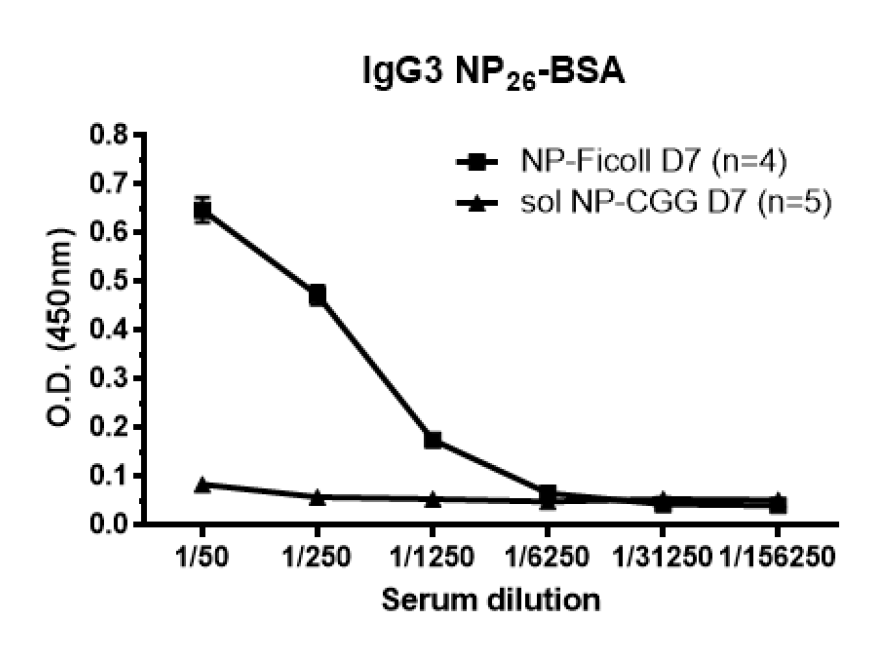

Supplement: S1 Fig — C57BL/6 mice were immunized with NP-Ficoll (square) or NP-CGG (triangle) on day 0. Serial serum dilution levels of anti-NP IgG3 Abs on NP26-BSA were measured by ELISA on day 7. Graphs show the mean ± SEM from at least n = 4 mice per group and the data are representative of one independent experiment. (TIF) [file pntd.0008358.s002.tif]

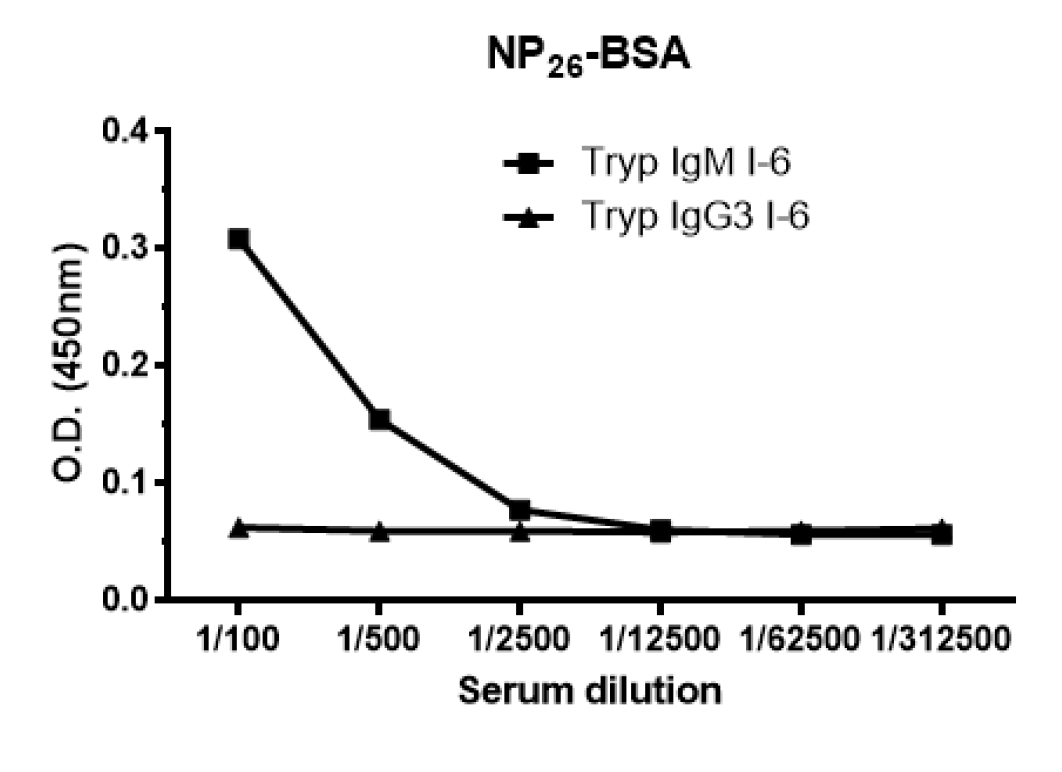

Supplement: S2 Fig — Serial serum dilution levels of anti-NP IgM (filled square) and IgG3 (filled triangle) Abs from pooled (n = 3) day 6-infected and uninfected mice were measured. The data are representative of two independent experiments. (TIF) [file pntd.0008358.s003.tif]
